# Who finds the road to palliative home care support? A nationwide analysis on the use of supportive measures for palliative home care using linked administrative databases

**DOI:** 10.1371/journal.pone.0213731

**Published:** 2019-03-12

**Authors:** Arno Maetens, Kim Beernaert, Luc Deliens, Birgit Gielen, Joachim Cohen

**Affiliations:** 1 Department of Family Medicine & Chronic Care, End-of-Life Care Research Group, Vrije Universiteit Brussel (VUB) & Ghent University, Brussels, Belgium; 2 Department of Public Health and Primary Care, Ghent University Hospital, Ghent, Belgium; 3 Intermutualistic Agency (IMA-AIM), Brussels, Belgium; Texas Technical University Health Sciences Center, UNITED STATES

## Abstract

**Background:**

Many countries developed supportive measures for palliative home care, such as financial incentives or multidisciplinary palliative home care teams. For policy makers, it is important to evaluate the use of these national palliative home care supportive measures on a population level.

**Methods and findings:**

Using routinely-collected data on all deaths in Belgium in 2012 (n = 107,847) we measured the use of four statutory supportive measures, specifically intended for patients who have obtained the legal palliative status, and three non-statutory supportive measures. Factors associated with uptake were analysed using multivariable logistic regression. Of all deaths of adult home-dwelling persons in Belgium (n = 87,007), 17.9 percent used at least one statutory supportive measure and 51.5 percent used at least one non-statutory supportive measure. In those who died of an illness indicative of palliative care needs 33.1 percent used at least one statutory supportive measure and 62.2 percent used at least one non-statutory supportive measure. Younger people and persons dying from cancer were more likely to use a statutory policy measure. Older people and persons dying from COPD were most likely to use a non-statutory policy measure. Women, non-single people, and those living in less urbanised areas were most likely to use any supportive measure.

**Conclusions:**

Statutory supportive measures for palliative home care are underused, even in a subpopulation of persons with potential palliative care needs. Policy makers should stimulate an equitable uptake, and reducing the observed inequalities is an important focus for health care policy.

## Introduction

Supportive measures for palliative home care exist in many countries [1,2]. Since the early 1990s, the development of these supportive measures was advocated by the World Health Organization to meet the growing need for quality palliative care at home, caused by ageing populations and increases in the number of people living with serious chronic illnesses[3–5]. Despite international differences in how they are financed and what criteria exist to access them, supportive measures for palliative home care generally exist in the form of financial incentives, supportive services, or workplace arrangements [6].

The use of hospice and specialist palliative care services has previously been studied in various countries[7–10]. A literature review from 2009 on patterns of access to community palliative care services concluded that patients with certain characteristics (e.g. younger, married, wealthier, and those with a caregiver at home) were more likely to access specialist palliative home care services [11]. However, these studies often used qualitative data or small sample sizes, had a constrained focus (e.g. only cancer patients, one specific palliative care service) or missed clarity in what they measure. In order to inform the public health policy debate and try to impact and improve the quality of care at the end-of-life on a population-level, however, it is important to measure and evaluate policies on that level [12].

Our study uses quality routinely collected data on all deaths in Belgium in 2012. We will describe the use of supportive measures for palliative home care in the full population of people who died while living at home, and in a population of those who died of an illness highly indicative of palliative care needs (i.e. neoplasms, COPD, other organ failures, neurodegenerative diseases and HIV/aids) [13,14]. Our research questions are: 1) what was in 2012 the frequency of the uptake of supportive measures for palliative home care; and 2) what sociodemographic and disease characteristics are associated with the uptake of these measures?

## Methods

### Design and setting

This retrospective observational study uses data from all individuals who died in 2012 in Belgium. Supportive measures for palliative home care exist in Belgium since 1985, and have since been further expanded [15]. Since 2002, palliative care is legally recognised as a right in Belgium.

### Data

Cohort data from eight routinely collected population-level databases, handled by three different organisations, were linked. The data consists of (1) the socio-demographic database of all individuals with healthcare insurance (legally mandatory in Belgium); (2) the health care database containing all reimbursed health care use data on home, nursing home, outpatient and hospital care; (3) the pharmaceutical database containing all reimbursed medication data; (4) Belgian Cancer Registry data with diagnostic information on all incidences of cancer including date of diagnosis and type of cancer; (5) death certificate data containing cause of death; (6) population registry data including nationality and household composition; (7) census data, including educational level and housing characteristics and (8) the fiscal database (including net taxable income).

After acquiring approvals from the relevant data protection agencies, the databases were linked for analysis in a secure and ethically responsible manner, guaranteeing the anonymity of the deceased. This process is described elsewhere [16].

### Subjects

To limit the analysis to those who can theoretically use the supportive measures, we excluded minors and people residing in a nursing home before death from the dataset.

To further limit the analysis to a population that is most likely to benefit from the measures, we made a second subpopulation of people who died from illnesses highly indicative of palliative care needs, defined by Rosenwax et al[14] as the ‘Minimal Estimate of potential users of palliative care’ through mixed-methods research, referred to here as the palliative subset. The following underlying causes of death were selected using the 10th revision of the International Statistical Classification of Diseases and Related Health Problems (ICD): neoplasms (ICD-10 C00-D48), chronic obstructive pulmonary disease (ICD-10 J40-44, J47), other organ failures (i.e. heart, renal, and liver failure) (ICD-10 I11-I13, I50, K70-72, N10-12, N18-19), neurodegenerative diseases (i.e. Alzheimer’s, Parkinson’s, motor neurone, and Huntington’s disease) (ICD-10 F01, F03, G10, G12, G20, G30), and HIV/aids (ICD-10 B20-24).

### Terms and classification of supportive measures

We defined supportive measures for palliative home care as: ‘all health care allowances and services (in addition to the standard primary care services) that can support the patient to remain at home in the last phase of life’. We divided supportive measures for palliative home care into statutory and non-statutory:

#### Statutory supportive measures for palliative home care

These supportive measures are specifically intended for patients who have obtained the legal ‘palliative status’, acquired after being diagnosed as: suffering from one or multiple irreversible diseases, progressing in an unfavourable direction, with serious physical/mental deterioration, where therapeutic and rehabilitative interventions no longer affect deterioration, the prognosis is poor and death is expected in the relatively short term (life expectancy of more than 24 hours and less than three months), having serious physical, psychological, social and existential needs requiring significant time-intensive and sustained support, and remaining or having the intention of dying at home [17]. The general practitioner of the patient (or another medical doctor) should formally make this diagnose to be able to receive these measures:

1. The use of a multi-disciplinary palliative home care team, which provides expertise to general practitioners, health professionals, counsellors, informal carers and volunteers. They consist of at least one team doctor, an administrative force and palliative experts (mainly nurses). It is free of charge and not limited in time;
2. Nursing care at home for patients with the palliative status. Palliative nurses are available to patients at home round-the-clock, have a basic training in palliative care, and need to add specific information to the nursing record (e.g. registration of symptoms, the pain scale). It is free of charge and not limited in time;
3. Physiotherapy at home for patients with the palliative status. It is free of charge and not limited in time;
4. The allowance for palliative home patients, a lump sum of € 647.16 (in 2012), obtainable twice and meant to cover for non-reimbursed costs related to the provision of palliative care in the home. The amount is fixed for all patients.

Apart from these measures, all out-of-pocket costs for general practitioner consultations are abolished for these patients.

#### Non-statutory supportive measures for palliative home care

1. Nursing care at home for heavily dependent home-patients. The out-of-pocket rate for heavily dependent patients depends on their level of care dependency (not free);
2. Physiotherapy at home for heavily dependent home-patients. The out-of-pocket rate for heavily dependent patients depends on their level of care dependency (not free);
3. The allowance for chronically ill patients, a lump sum of up to €576.10 (in 2012) for heavily care-dependent people who have exceeded a certain amount of out-of-pocket costs for care in two consecutive years. The amount is linked to the level of care dependency (divided into three levels).

### Sociodemographic characteristics

Population characteristics available through the datasets are: age at death, sex, nationality, household type, housing comfort, educational level (using UNESCO’s International Standard Classification of Education (ISCED)), region, degree of urbanisation, annual personal net taxable income and underlying cause of death (using the 10^th^ revision of the International Classification of Diseases (ICD-10), categorized as: neoplasms, COPD, other organ failure, neurodegenerative disease, HIV/aids, other). For codification clarifications and detailed information on data administrators and data sources see S1 Appendix.

### Data analysis

Descriptive statistics were used to describe population characteristics in the full population and in the palliative subset. To compare the chances of receiving a policy measure to support palliative home care between different population groups a multivariable binary logistic regression was performed with uptake (of a statutory measure, a non-statutory measure, or one or more of all measures) as the dependent variable. A hierarchical approach was followed to build the model (see S1 Appendix). An alternative model was analysed on a subgroup of only cancer deaths (see S1 Table). The analyses were generated using the SAS Enterprise Guide 7.1 software.

### Ethics approvals

This study received approvals by the Commission for Medical Ethics of the University Hospital Brussels (B.U.N. 143201629410). Trusted Third Parties (TTPs) ‘eHealth’ and ‘Crossroads Bank for Social Security (CBSS)’ were responsible for the deidentification process and deterministic one-to-one record linkage of the databases. In accordance with Belgian law, approvals for access to and integration of the various databases were obtained from two separate national sectoral committees for privacy protection: the ‘Sectoral Committee of Social Security and Health, Section Health’ and the ‘Statistical Supervisory Committee’. Both are subcommittees of the Belgian Commission for the Protection of Privacy. No informed consent was required. For a full description of the data linkage procedure, including details on the data anonymization, see Maetens et al.[16].

## Results

### Population characteristics

In 2012, 87,007 health-insured adult persons died in Belgium while living at home (Table 1). Of these, 38,657 died from a cause indicative of palliative care need: 29.5% died from a neoplasm, 4.3% from COPD, 5.6% from another organ failure, 5% from neurodegenerative disease, and 0.04% from HIV/aids.

**Table 1:** Characteristics of all deaths and deaths of persons with potential palliative care needs (in %).

	All deaths	Palliative subset
*Number*	87,007	38,657
*Age*		
18–64	19.3	18.7
65–74	17.0	20.3
75–84	31.6	33.3
85–94	29.0	25.5
94+	3.2	2.2
*Mean age (SD)*	*76*.*18 (14*.*00)*	*75*.*92 (12*.*36)*
*Gender*		
Man	54.7	55.6
Woman	45.3	44.4
*Underlying cause of death*		
Neoplasms	29.5	66.4
Other organ failure	5.6	9.4
COPD	4.3	9.6
Neurodegenerative disease	5.0	11.3
HIV/aids	0.04	3.3
*Nationality*		
Belgian	93.7	94.9
Non-Belgian	6.3	5.1
*Household composition*		
Single person household	38.7	34.6
Married	47.6	52.6
Living together	4.3	4.1
One-parent family	6.6	6.1
Other	3.0	2.7
*Missing*^*a*^	*1*.*6*	*0*.*2*
*Housing standard*^*b*^		
High	44.9	48.0
Moderate	18.8	17.2
Low	26.8	26.3
Below basic level	9.6	8.5
*Missing*^*a*^	*9*.*4*	*7*.*7*
*Education level*		
No primary education	8.6	8.1
Primary school education	34.8	34.7
Secondary school education	45.1	45.0
Post-secondary school education	11.5	12.2
*Missing*^*a*^	*13*.*1*	*11*.*1*
*Income level*		
Q1 (lowest to €12,221)	26.7	27.0
Q2: (€12,222 to €14,497)	23.2	22.5
Q3: (€14,498 to €18,346)	24.4	24.7
Q4: (18,347 to highest)	25.7	25.7
*Missing*^*a*^	*4*.*1*	*2*.*1*
*Region*		
Brussels Capital region	9.2	8.3
Walloon region	35.7	32.8
Flemish region	55.1	59.0
*Missing*^*a*^	*1*.*9*	*0*.*7*
*Urbanisation*^*c*^		
Very high	32.2	30.8
High	27.7	28.3
Average	25.6	27.0
Low	13.1	12.5
Rural	1.5	1.4
*Missing*^*a*^	*1*.*9*	*0*.*7*

^*a*^All percentages are presented as valid percentages. Percentages for missing values are calculated separately.

^*b*^ Housing standard was operationalised by the national bureau for statistics and was based on several criteria of comfort, e.g. ‘having a toilet and bathroom with bath and/or shower’, ‘having central heating’, ‘having a kitchen of min. 4m2’. For the detailed operationalisation, see S1 Appendix.

^c^ Degree of urbanisation was operationalised by the national bureau for statistics and was based on morphological and functional level of urbanisation (strong or weak). For the detailed operationalisation, see S1 Appendix.

### Uptake of supportive measures for palliative home care

Of all home-dwelling adults, 17.9% used a statutory measure, 51.5% a non-statutory measure, 55.2% at least one supportive measure, and 14.1% at least one statutory and non-statutory measure (Table 2).

**Table 2:** The use of supportive measures for palliative home care (in %).

	All deaths*	Palliative subset
*Number (n)*	87,007	38,657
*Statutory palliative home care measures*	*17*.*9*	*33*.*1*
Allowance for palliative home patients	16.0	30.4
Multi-disciplinary support team	8.6	17.1
Nursing care for palliative home patients	12.7	23.7
Physiotherapy for palliative home patients	4.0	7.1
*Non-statutory palliative home care measures*	*51*.*5*	*62*.*2*
Nursing care for heavily dependent persons	29.7	34.6
Physiotherapy for heavily dependent persons	24.4	25.4
Allowance for chronically ill patients	29.2	38.4
*Used one of the above measures*	*55*.*2*	*69*.*7*
*Used a ‘statutory’ and a ‘non-statutory’ measure*	14.1	25.7

Patients were able to receive several measures at once, thus numbers of uptake do not add up.

*All deaths of adult persons not residing in a nursing home in the last year of life.

Of those in the palliative subset, 33.1% used a statutory and 62.2% a non-statutory measure; 69.7% used at least one of each, and 25.7% used both (see S2 Table). The statutory and non-statutory measures were used by 42.7% and 62.3% respectively of those dying of neoplasms, 11.4% and 65.6% of COPD, 13.1% and 61.7% of other organ failures, 17.7% and 59.8% of neurodegenerative diseases, and 5.9% and 32.4% of HIV/aids.

### Characteristics associated with uptake of a supportive measure for palliative home care

Multivariable logistic analysis for the palliative subset indicated that those under 65 years were significantly more likely to use a statutory measure compared to those above 95 years old (Odds Ratio = 1.32; 95% Confidence Interval [1.07–1.62]), but not a non-statutory measure (Table 3). The age groups between 65 years old and 94 years old were not significantly more or less likely to use a statutory measure compared to those aged over 95, but significantly more likely to use a non-statutory measure. Age was not a significant predictor for the use of either a statutory or non-statutory measure (third model). Women were more likely to use a statutory (OR = 1.10[1.04–1.16]) or non-statutory (OR = 1.35[1.28–1.41]) measure than men. Compared with those who died from neoplasms, people who died from COPD (OR = 0.18[0.16–0.20]), other organ failures (OR = 0.22[0.20–0.24]), neurodegenerative disease (OR = 0.30[0.27–0.33]), or HIV/aids (OR = 0.17[0.04–0.72]) were less likely to use a statutory measure. Compared with people who died from neoplasms, those who died from COPD were more likely to use a non-statutory measure (OR = 1.22[1.13–1.32]), but those who died from neurodegenerative diseases (OR = 0.86[0.80–0.92]) or from HIV/aids (OR = 0.41[0.18–0.94]) were less likely to receive such a measure. Results predicting the use of a non-statutory measure were not significant for those who died from other organ failure.

**Table 3:** Factors associated with the use of supportive measures for palliative home care in the palliative subset population (n = 32,075).

	Statutory		Non-statutory		Statutory or non-statutory	
	OR	95% CI	OR	95% CI	OR	95% CI
Age						
18–64	**1.32**	(1.07–1.62)	1.09	(0.93–1.28)	-	-
65–74	1.19	(0.97–1.47)	**1.20**	(1.02–1.40)	-	-
75–84	1.10	(0.89–1.34)	**1.22**	(1.05–1.43)	-	-
85–94	0.97	(0.79–1.20)	**1.19**	(1.02–1.39)	-	-
94+	ref	-	ref	-	ref	-
Sex						
Male	ref	-	ref	-	ref	-
Female	**1.10**	(1.04–1.16)	**1.35**	(1.28–1.41)	**1.3**	(1.23–1.37)
Cause of death						
Neoplasm	ref	-	ref	-	ref	-
Other organ failure	**0.22**	(0.20–0.24)	1.0	(0.93–1.08)	**0.68**	(0.63–0.73)
COPD	**0.18**	(0.16–0.20)	**1.22**	(1.13–1.32)	0.82	(1.75–0.89)
Neurodegenerative disease	**0.30**	(0.27–0.33)	**0.86**	(0.80–0.92)	**0.61**	(0.56–0.66)
HIV/aids	**0.17**	(0.04–0.72)	**0.41**	(0.18–0.94)	**0.36**	(0.15–0.87)
Household composition						
Single person household	ref	-	ref	-	ref	-
Married	**1.76**	(1.65–1.87)	**1.64**	(1.55–1.73)	**1.75**	(1.65–1.86)
Living together	**1.42**	(1.24–1.62)	**1.28**	(1.14–1.44)	**1.34**	(1.18–1.53)
One-parent family	**1.35**	(1.21–1.52)	**1.24**	(1.12–1.36)	**1.27**	(1.14–1.41)
Other	**1.40**	(1.18–1.65)	**1.25**	(1.08–1.44)	**1.35**	(1.15–1.58)
Housing standard						
Below low	ref	-	ref	-	ref	-
High	**1.28**	(1.16–1.42)	-	-	**1.37**	(1.25–1.51)
Average	1.10	(0.98–1.23)	-	-	**1.15**	(1.04–1.28)
Low	**1.14**	(1.02–1.27)	-	-	**1.19**	(1.09–1.31)
Education level						
No education	ref	-	ref	-	ref	-
Primary school education	-	-	0.99	(0.90–1.08)	0.94	(0.86–1.04)
Lower secondary school education	-	-	1.05	(0.96–1.14)	0.97	(0.88–1.07)
Post-secondary school education	-	-	**1.22**	(1.10–1.36)	**1.13**	(1.00–1.27)
Income level						
Q1 (lowest)	ref	-	ref	-	ref	-
Q2	1.06	(0.99–1.15)	1.05	(0.99–1.13)	**1.08**	(1.01–1.16)
Q3	1.01	(0.94–1.09)	**1.13**	(1.06–1.21)	**1.12**	(1.01–1.21)
Q4 (highest)	0.93	(0.87–1.00)	**1.15**	(1.07–1.23)	1.06	(0.99–1.14)
Region						
Brussels-capital region	ref	-	ref	-	ref	-
Walloon region	**1.14**	(1.01–1.29)	**1.17**	(1.06–1.29)	**1.15**	(1.03–1.27)
Flemish region	**1.73**	(1.54–1.94)	**1.15**	(1.04–1.26)	**1.33**	(1.20–1.46)
Urbanisation						
Very high	ref	-	ref	-	ref	-
High	**1.17**	(1.09–1.26)	**1.1**	(1.04–1.17)	**1.1**	(1.03–1.18)
Average	**1.39**	(1.29–1.49)	**1.31**	(1.23–1.40)	**1.34**	(1.25–1.44)
Low	**1.47**	(1.34–1.61)	**1.28**	(1.17–1.39)	**1.32**	(1.21–1.45)
Rural	**1.60**	(1.29–1.99)	**1.26**	(1.03–1.54)	**1.32**	(1.06–1.65)

Reference category is presented on the left. Exploratory variables that did not significantly add to the model were excluded in the final model; models were built in a stepwise manner. CI = Confidence Interval. *P*-value < 0.05 in bold.

People who were married (OR = 1.75[1.65–1.86]), living together (OR = 1.34[1.18–1.53]), forming a one-parent family (OR = 1.27[1.14–1.41]), or being part of another sort of household (OR = 1.35[1.15–1.58]) were more likely to use at least one of all supportive measures than those living alone. People living in the Flemish (OR = 1.33[1.20–1.46]) or in the Walloon region (OR = 1.15[1.03–1.27]) were more likely to receive at least one of all measures than those living in the Brussels Capital region. People living in an area with a lower degree of urbanisation were more likely to use a supportive measure than those in an area with a higher degree of urbanisation.

Educational level was found to be a significant predictor of supportive measure use only in the group with post-secondary school education, and only for the use of a non-statutory measure (OR = 1.22[1.10–1.36]) or for any supportive measures (OR = 1.13[1.00–1.27]). Having higher standard housing is a significant predictor of statutory measure use, of any measure use, but not of non-statutory measure use. Nationality was included in the analyses but was found not to be significant.

## Discussion

Using linked data of all deaths in Belgium in 2012, this study found that the uptake of supportive measures for palliative home care in a population with potential palliative care needs was relatively low. Only a third used a statutory measure (33.1%). The use of non-statutory measures was found to be more frequent in this population (62.2%). Statutory measures were more likely to be used by younger people and those with neoplasms, non-statutory measures by older people and those dying of COPD.

### Strengths and limitations

To our knowledge, this is the first study to measure uptake of both statutory and non-statutory supportive measures for palliative home care on a population-level. In Belgium, membership to one of the seven national health insurers is mandatory for the full population, thus the data cover (nearly) all individuals. However, additional private insurance is possible, which is not included in our data. We were able to measure multiple types of supportive measures, such as financial incentives, supportive home care services and multidisciplinary support teams. The use of linked data for all Belgian citizens for a given year from routinely-collected administrative, disease-specific and insurance databases offers a rich, powerful and reliable resource for studying important public health issues [18,19]. Our data also has its limitations. Although the identification of people in the palliative subset is based on a previously validated and frequently used estimation, the estimation is based on underlying cause of death, not on an actually observed or stated palliative care need[14]. Reimbursement data also do not capture other forms of support for palliative home care (e.g. care given by informal caregivers, services organised outside reimbursement schemes) or social support (e.g. religious based, kinship networks).

### Interpretation of results

Only a third of home-dwelling people with potential palliative care needs made use of a statutory supportive measure for palliative home care. This low uptake is possibly related to the legal criteria surrounding these measures; in order to be granted legal palliative home patient status, life expectancy must be estimated as ‘more than 24 hours and less than three months’ [17]. Firstly, prognostication of survival in patients with advanced cancer has been well-researched, showing consistently that both physicians and patients are unable to correctly predict their life expectancy, most commonly overestimating it [20–23]. The disease trajectories of non-cancer serious illnesses are even more unpredictable, further complicating the process of prognostication [24–26]. Secondly, people confronted with non-cancer illnesses like organ failure or dementia often see their illness as ‘a way of life’[27] rather than a life-threatening illness, therefore possibly not perceiving themselves as having palliative home care needs [28–30]. The possibility of receiving a statutory supportive measure can thus come too late or even not at all. This hypothesis is supported by findings of a previous study that focussed on the physician’s reasons for not referring people with life-limiting illnesses to specialist palliative care services in Belgium in which physicians mentioned ‘not [having] enough time to initiate palliative care’ as one of the three major reasons for non-referral [13]. Indeed, recent survey data from Flanders shows that specialised palliative care is initiated with a median of only twenty days before death [31]. Our finding that younger people and those who died of neoplasms were most likely to use statutory supportive measures confirms previous findings [11], and could be related to the problems of recognising the terminal phase, something frequently reported to be more difficult in older population groups with chronic illnesses other than cancer [32,33].

The uptake of non-statutory supportive measures was higher than statutory supportive measures. The criteria for these non-statutory supportive measures are based on high care needs combined with high out-of-pocket costs for two consecutive years, and not on life expectancy, which could explain why they are used more frequently than statutory measures [34–36]. This could also explain our finding that the chance of using a non-statutory measure was highest among people who died of COPD, who are prone to high care needs and out-of-pocket costs [37,38]. It remains unclear, however, whether statutory and non-statutory measures contribute equally to the effective support of palliative home care.

The uptake of supportive measures for palliative home care seems to be influenced, apart from aspects related to prognosis, by factors such as social support and socio-economic position. The higher chances of using statutory and non-statutory measures in those living with others compared with single-person households suggests that the presence of social support in the household is an important factor in organising palliative home care and using supportive measures to do so. The influence of socioeconomic characteristics on the knowledge or use of palliative care has very rarely been studied, and with differing results [39–41]. Our findings partly suggest a social gradient exists in the use of supportive measures; the lower likelihood of using non-statutory measures in the lowest income category can indicate inequality. This result differs from the uptake of statutory measures, however, where people in the highest income category were significantly less likely to use these measures when compared with the lowest income category. The better chances of people with post-secondary education receiving a non-statutory measure may reflect differences in social capital that give them an advantage in knowing about and obtaining the measures. The advantage of those living in a home with a high standard of housing compared with those with a lower standard is an additional indication that there is some social inequality.

It seems likely that knowledge of existing supportive measures also plays a role. Because palliative care in Belgium has traditionally focussed mainly on cancer patients [42], their advantage in using statutory supportive measures could be due to both oncologists and patients being better informed. We also found that people living in Flanders were more likely to use a supportive measure than those in the Brussels Capital region. This may be explained by regional differences in general information provision and knowledge of palliative care or to differences in the social environment (e.g. lower social fragmentation in more rural areas) and national healthcare policy (concentration of large academic hospitals in metropolitan areas creating a pull factor towards end-of-life care in hospitals) [43].

### Suggestions for further research and policy recommendations

Our study is a first step in understanding who makes use of supportive measures for palliative home care, and who is missing out on them. Future research should focus on how inequalities in the uptake of these measures influence different outcomes, such as place of death, quality of care, and costs at the end of life. Additionally, differences in the impact of using statutory or non-statutory measures on these outcomes should be compared. In this way, public health policy can be quantitatively evaluated and further improved. Qualitative research also needs to further examine the motivation and reasons behind the differences in uptake between certain population groups, and the differences in uptake between statutory and non-statutory supportive measures, as these are not registered in administrative data.

The large gap in uptake between statutory and non-statutory supportive measures among a subgroup of people in potential palliative care need suggests that there remains inequity in accessing statutory measures. Policy-makers should focus on including underreached groups, such as older people and those with non-cancer illnesses. The life expectancy criterion to qualify for the statutory supportive measures should be re-evaluated. This is possibly a major barrier that prevents many patients with palliative care needs to make use of them, in particular older patients and those with a non-cancer diagnosis.

## Conclusion

A relatively large proportion of people who are dying use some supportive measure for palliative home care. However, the measures specifically intended to support palliative home care are underused, even in a subpopulation of those who die of an illness indicative of palliative care needs. Stimulating an equitable uptake of supportive measures intended to support palliative home care, and reducing the observed social inequalities in their uptake, is an important focus for health care policy.

### Data availability statement

Due to ethical concerns with regard to sensitive and potentially identifying data, the supporting data cannot be made openly available, as stated by the Sectoral Committee of Social Security and Health—Department Health and the Data Protection Authority. Data availability and access regulations are available upon request with the separate database administrators. The InterMutualistic Agency can be contact via the website (https://ima-aim.be), Statistics Belgium via e-mail (statbel@economie.fgov.be), Belgian Cancer Registry via e-mail (info@kankerregister.org).

## Supporting information

S1 Table Factors associated with the use of supportive measures for palliative home care among a subgroup of cancer deaths (n = 21,530). (DOCX)

S2 Table Uptake of policy measures to support palliative care at home by characteristics of the palliative subset (row %) (n = 38,657). (DOCX)

S1 Appendix Supplementary information on the operationalisation of variables. (DOCX)

## References

[1] Gori C, Fernandez J-L. Long-term Care Reforms in OECD Countries. Policy Press; 2015.

[2] Woitha K, Carrasco JM, Clark D, Lynch T, Garralda E, Martin-Moreno JM, et al Policy on palliative care in the WHO European region: an overview of progress since the Council of Europe’s (2003) recommendation 24. Eur J Public Health 2015:ckv201. 10.1093/eurpub/ckv201 26545804

[3] Davies E, Higginson IJ, editors. Palliative care. The solid facts 2004.

[4] Stjernswärd J, Foley KM, Ferris FD. The Public Health Strategy for Palliative Care. J Pain Symptom Manage 2007;33:486–93. 10.1016/j.jpainsymman.2007.02.016 17482035

[5] European Commission. Long-term care in the European Union 2008.

[6] Maetens A, Beernaert K, Deliens L, Aubry R, Radbruch L, Cohen J. Policy Measures to Support Palliative Care at Home: A Cross-Country Case Comparison in Three European Countries. J Pain Symptom Manage 2017;54:523–529.e5. 10.1016/j.jpainsymman.2017.07.022 28736105

[7] Beccaro M, Costantini M, Merlo DF, ISDOC Study Group. Inequity in the provision of and access to palliative care for cancer patients. Results from the Italian survey of the dying of cancer (ISDOC). BMC Public Health 2007;7:66 10.1186/1471-2458-7-66 17466064 PMC1885253

[8] Jakobsson E, Bergh I, Öhlén J, Odén A, Gaston-Johansson F. Utilization of health-care services at the end-of-life. Health Policy 2007;82:276–87. 10.1016/j.healthpol.2006.10.003 17097757

[9] Pivodic L, Pardon K, Block LV den, Casteren VV, Miccinesi G, Donker GA, et al Palliative Care Service Use in Four European Countries: A Cross-National Retrospective Study via Representative Networks of General Practitioners. PLOS ONE 2013;8:e84440 10.1371/journal.pone.0084440 24386381 PMC3875565

[10] Willemse E, Anthierens S, Farfan-Portet MI, Schmitz O, Macq J, Bastiaens H, et al Do informal caregivers for elderly in the community use support measures? A qualitative study in five European countries. BMC Health Serv Res 2016;16:270 10.1186/s12913-016-1487-2 27423182 PMC4947246

[11] Walshe C, Todd C, Caress A, Chew-Graham C. Patterns of Access to Community Palliative Care Services: A Literature Review. J Pain Symptom Manage 2009;37:884–912. 10.1016/j.jpainsymman.2008.05.004 19097748

[12] Cohen J, Deliens L. A Public Health Perspective on End of Life Care. Oxford University Press; 2012.

[13] Beernaert K, Deliens L, Pardon K, Van den Block L, Devroey D, Chambaere K, et al What Are Physicians’ Reasons for Not Referring People with Life-Limiting Illnesses to Specialist Palliative Care Services? A Nationwide Survey. PloS One 2015;10:e0137251 10.1371/journal.pone.0137251 26356477 PMC4565578

[14] Rosenwax LK, McNamara B, Blackmore AM, Holman CDJ. Estimating the size of a potential palliative care population. Palliat Med 2005;19:556–62. 10.1191/0269216305pm1067oa 16295289

[15] Houttekier D, Cohen J, Van den Block L, Bossuyt N, Deliens L. Involvement of palliative care services strongly predicts place of death in Belgium. J Palliat Med 2010;13:1461–8. 10.1089/jpm.2010.0279 21133810

[16] Maetens A, De Schreye R, Faes K, Houttekier D, Deliens L, Gielen B, et al Using linked administrative and disease-specific databases to study end-of-life care on a population level. BMC Palliat Care 2016;15:86 10.1186/s12904-016-0159-7 27756296 PMC5069861

[17] Keirse M, Thibo T. Evaluatierapport palliatieve zorg. 2014.

[18] Jutte DP, Roos LL, Brownell MD. Administrative Record Linkage as a Tool for Public Health Research In: Fielding JE, Brownson RC, Green LW, editors. Annu. Rev. Public Health Vol 32, vol. 32, Palo Alto: Annual Reviews; 2011, p. 91–108. 10.1146/annurev-publhealth-031210-100700 21219160

[19] Field K, Kosmider S, Johns J, Farrugia H, Hastie I, Croxford M, et al Linking data from hospital and cancer registry databases: should this be standard practice? Intern Med J 2010;40:566–73. 10.1111/j.1445-5994.2009.01984.x 19460066

[20] Clarke MG, Ewings P, Hanna T, Dunn L, Girling T, Widdison AL. How accurate are doctors, nurses and medical students at predicting life expectancy? Eur J Intern Med 2009;20:640–4. 10.1016/j.ejim.2009.06.009 19782929

[21] Allen LA, Yager JE, Funk MJ, Levy WC, Tulsky JA, Bowers MT, et al Discordance Between Patient-Predicted and Model-Predicted Life Expectancy Among Ambulatory Patients With Heart Failure. JAMA 2008;299:2533–42. 10.1001/jama.299.21.2533 18523222 PMC3623529

[22] Glare P, Virik K, Jones M, Hudson M, Eychmuller S, Simes J, et al A systematic review of physicians’ survival predictions in terminally ill cancer patients. BMJ 2003;327:195 10.1136/bmj.327.7408.195 12881260 PMC166124

[23] White N, Reid F, Harris A, Harries P, Stone P. A Systematic Review of Predictions of Survival in Palliative Care: How Accurate Are Clinicians and Who Are the Experts? PloS One 2016;11:e0161407 10.1371/journal.pone.0161407 27560380 PMC4999179

[24] Coventry PA, Grande GE, Richards DA, Todd CJ. Prediction of appropriate timing of palliative care for older adults with non-malignant life-threatening disease: a systematic review. Age Ageing 2005;34:218–27. 10.1093/ageing/afi054 15863407

[25] Brandt HE, Ooms ME, Ribbe MW, van der Wal G, Deliens L. Predicted survival vs. actual survival in terminally ill noncancer patients in Dutch nursing homes. J Pain Symptom Manage 2006;32:560–6. 10.1016/j.jpainsymman.2006.06.006 17157758

[26] Lau F, Cloutier-Fisher D, Kuziemsky C, Black F, Downing M, Borycki E, et al A systematic review of prognostic tools for estimating survival time in palliative care. J Palliat Care 2007;23:93–112. 17853845

[27] Landers A, Wiseman R, Pitama S, Beckert L. Patient perceptions of severe COPD and transitions towards death: a qualitative study identifying milestones and developing key opportunities. NPJ Prim Care Respir Med 2015;25:15043 10.1038/npjpcrm.2015.43 26158886 PMC4497313

[28] Carpenter JG, Berry PH, Ersek M. Nursing home care trajectories for older adults following in-hospital palliative care consultation. Geriatr Nurs N Y N 2017;38:531–6. 10.1016/j.gerinurse.2017.03.016 28457493 PMC5659976

[29] Dempsey D. Advance care planning for people with dementia: benefits and challenges. Int J Palliat Nurs 2013;19:227–34. 10.12968/ijpn.2013.19.5.227 23971306

[30] Gysels M, Higginson IJ. Access to services for patients with chronic obstructive pulmonary disease: the invisibility of breathlessness. J Pain Symptom Manage 2008;36:451–60. 10.1016/j.jpainsymman.2007.11.008 18495412

[31] Moreels S, Van den Block L, Pivodic L, Boffin N, Penders Y, Van Casteren V. Zorg aan het levenseinde in het Vlaamse gewest in de periode 2005–2014 2016.

[32] Solano JP, Gomes B, Higginson IJ. A Comparison of Symptom Prevalence in Far Advanced Cancer, AIDS, Heart Disease, Chronic Obstructive Pulmonary Disease and Renal Disease. J Pain Symptom Manage 2006;31:58–69. 10.1016/j.jpainsymman.2005.06.007 16442483

[33] Field MJ, Cassel CK, Institute of Medicine (U.S.), Committee on Care at the End of Life. Approaching death: improving care at the end of life. Washington, D.C.: National Academy Press; 1997. 25121204

[34] Teno JM, Weitzen S, Fennell ML, Mor V. Dying Trajectory in the Last Year of Life: Does Cancer Trajectory Fit Other Diseases? J Palliat Med 2001;4:457–64. 10.1089/109662101753381593 11798477

[35] Murray SA, Kendall M, Boyd K, Sheikh A. Illness trajectories and palliative care. BMJ 2005;330:1007–11. 10.1136/bmj.330.7498.1007 15860828 PMC557152

[36] Murtagh FEM, Preston M, Higginson I. Patterns of dying: palliative care for non-malignant disease. Clin Med 2004;4:39–44. 10.7861/clinmedicine.4-1-39 14998265 PMC4954272

[37] Faes K, Frène VD, Cohen J, Annemans L. Resource Use and Health Care Costs of COPD Patients at the End of Life: A Systematic Review. J Pain Symptom Manage 2016;52:588–99. 10.1016/j.jpainsymman.2016.04.007 27401511

[38] Emanuel EJ, Ash A, Yu W, Gazelle G, Levinsky NG, Saynina O, et al Managed Care, Hospice Use, Site of Death, and Medical Expenditures in the Last Year of Life. Arch Intern Med 2002;162:1722–8. 10.1001/archinte.162.15.1722 12153375

[39] Hanratty B, Jacoby A, Whitehead M. Socioeconomic differences in service use, payment and receipt of illness-related benefits in the last year of life: findings from the British Household Panel Survey. Palliat Med 2008;22:248–55. 10.1177/0269216307087140 18477719

[40] Gisquet E, Julliard S, Geoffroy-Perez B. Do social factors affect the place of death? Analysis of home versus institutional death over 20 years. J Public Health Oxf Engl 2015 10.1093/pubmed/fdv167 28158559

[41] Koffman J, Burke G, Dias A, Raval B, Byrne J, Gonzales J, et al Demographic factors and awareness of palliative care and related services. Palliat Med 2007;21:145–53. 10.1177/0269216306074639 17344263

[42] World Health Organization. Palliative care. The solid facts 2004.

[43] Cohen J, Chambaere K, Bilsen J, Houttekier D, Mortier F, Deliens L. Influence of the metropolitan environment on end-of-life decisions: A population-based study of end-of-life decision-making in the Brussels metropolitan region and non-metropolitan Flanders. Health Place 2010;16:784–93. 10.1016/j.healthplace.2010.04.003 20430683

