# Supplementary material for: Who finds the road to palliative home care support? A nationwide analysis on the use of supportive measures for palliative home care using linked administrative databases
Source: PLoS One. 2019 Mar 12;14(3):e0213731. doi: 10.1371/journal.pone.0213731 (PMC6414004; doi:10.1371/journal.pone.0213731)
Supplement: S1 Table — (DOCX) [file pone.0213731.s001.docx]

**Table S1: Factors associated with the use of supportive measures for palliative home care among a subgroup of cancer deaths (n=21,530)**

|  | **Statutory** | | **Non-statutory** | | **Statutory or non-statutory** | |
| --- | --- | --- | --- | --- | --- | --- |
|  | OR | 95% CI | OR | 95% CI | OR | 95% CI |
| Age |  |  |  |  |  |  |
| 18-64 | **1,97** | (1,42-2,74) | - | - | - | - |
| 65-74 | **1,74** | (1,25-2,42) | - | - | - | - |
| 75-84 | **1,65** | (1,19-2,29) | - | - | - | - |
| 85-94 | 1,36 | (0,98-1,89) | - | - | - | - |
| 95+ | ref | - | ref | - | ref | - |
| Sex |  |  |  |  |  |  |
| Male | ref | - | ref | - | ref | - |
| Female | **1,09** | (1,02-1,15) | **1,50** | (1,41-1,59) | **1,42** | (1,33-1,52) |
| Household composition |  |  |  |  |  |  |
| Single person household | ref | - | ref | - | ref | - |
| Married | **1,73** | (1,62-1,85) | **1,64** | (1,53-1,75) | **1,81** | (1,68-1,95) |
| Living together | **1,36** | (1,18-1,57) | **1,29** | (1,12-1,49) | **1,39** | (1,19-1,63) |
| One-parent family | **1,27** | (1,12-1,45) | 1,12 | (0,99-1,27) | **1,18** | (1,03-1,35) |
| Other | **1,33** | (1,09-1,62) | 1,11 | (0,91-1,34) | 1,20 | (0,97-1,49) |
| Housing standard |  |  |  |  |  |  |
| Below low | ref | - | ref | - | ref | - |
| High | **1,27** | (1,13-1,43) | - | - | **1,43** | (1,26-1,61) |
| Average | 1,02 | (0,89-1,16) | - | - | 1,13 | (0,99-1,29) |
| Low | **1,13** | (1,00-1,28) | - | - | **1,18** | (1,04-1,33) |
| Education level |  |  |  |  |  |  |
| No education | ref | - | ref | - | ref | - |
| Primary school education | **0,89** | (0,79-0,99) | 1,05 | (0,94-1,17) | 0,97 | (0,86-1,10) |
| Lower secondary school education | **0,84** | (0,75-0,94) | 1,10 | (0,98-1,22) | 0,99 | (0,88-1,12) |
| Post-secondary school education | **0,86** | (0,76-0,98) | **1,29** | (1,13-1,46) | **1,16** | (1,00-1,35) |
| Income level |  |  |  |  |  |  |
| Q1 (lowest) | ref | - | ref | - | ref | - |
| Q2 | - | - | 1,08 | (0,99-1,17) | **1,37** | (1,04-1,81) |
| Q3 | - | - | **1,24** | (1,15-1,35) | **1,21** | (1,11-1,33) |
| Q4 (highest) | - | - | **1,30** | (1,20-1,41) | **1,18** | (1,07-1,29) |
| Region |  |  |  |  |  |  |
| Brussels-capital region | ref | (0,94-1,23) | ref | - | ref | - |
| Walloon region | **1,07** | (1,54-1,99) | - | - | 1,05 | (0,92-1,20) |
| Flemish region | **1,75** | (1,08-1,26) | - | - | **1,30** | (1,14-1,47) |
| Urbanisation |  |  |  |  |  |  |
| Very high | ref | - | ref | - | ref | - |
| High | **1,17** | (1,08-1,26) | **1,15** | (1,07-1,23) | **1,15** | (1,07-1,23) |
| Average | **1,38** | (1,28-1,49) | **1,33** | (1,24-1,43) | **1,13** | (1,04-1,23) |
| Low | **1,44** | (1,29-1,59) | **1,36** | (1,24-1,49) | **1,36** | (1,25-1,49) |
| Rural | **1,59** | (1,24-2,04) | **1,32** | (1,04-1,69) | **1,38** | (1,23-1,55) |
