# Supplementary material for: Who finds the road to palliative home care support? A nationwide analysis on the use of supportive measures for palliative home care using linked administrative databases
Source: PLoS One. 2019 Mar 12;14(3):e0213731. doi: 10.1371/journal.pone.0213731 (PMC6414004; doi:10.1371/journal.pone.0213731)
Supplement: S2 Table — (DOCX) [file pone.0213731.s002.docx]

S2 Table. Uptake of policy measures to support palliative care at home by characteristics of the palliative subset (row %) (n=38,657)

|  | Statutory palliative home care policy measures | | | | | | Non-statutory palliative home care policy measures | | | |  |
| --- | --- | --- | --- | --- | --- | --- | --- | --- | --- | --- | --- |
|  | Any statutory measure | | Allowance for palliative home patients | Multi-disciplinary support team | Nursing care for palliative home patients | Physiotherapy for palliative home patients | Any non-statutory measure | Allowance for chronic patients | Nursing care at home | Physiotherapy at home | Any measure |
| *Number* | *12,806* | | *11,736* | *6,592* | *9,165* | *2,724* | *24,050* | *14,843* | *13,379* | *9,823* | *26,941* |
| Age |  | |  |  |  |  |  |  |  |  |  |
| 18-64 | 42.6 | | 40.4 | 25.3 | 29.5 | 9.4 | 61.1 | 49.8 | 20.2 | 16.8 | 71.3 |
| 65-74 | 38.7 | | 36.2 | 20.8 | 27.8 | 8.6 | 63.6 | 47.0 | 28.3 | 22.0 | 72.2 |
| 75-84 | 32.5 | | 29.7 | 15.8 | 23.7 | 6.8 | 63.5 | 38.2 | 37.8 | 28.8 | 70.9 |
| 85-94 | 23.7 | | 20.6 | 10.7 | 17.0 | 4.7 | 60.8 | 25.3 | 45.1 | 30.1 | 65.8 |
| 95+ | 18.3 | | 15.4 | 5.8 | 14.5 | 4.6 | 55.2 | 16.0 | 46.9 | 24.7 | 58.8 |
| Gender |  | |  |  |  |  |  |  |  |  |  |
| Male | 34.2 | | 31.9 | 17.4 | 24.4 | 6.9 | 60.8 | 38.7 | 32.9 | 23.5 | 69.1 |
| Female | 31.8 | | 28.5 | 16.6 | 22.9 | 7.3 | 64.0 | 38.1 | 36.8 | 27.8 | 70.5 |
| Cause of death | |  |  |  |  |  |  |  |  |  |  |
| Neoplasms | | 42.7 | 39.9 | 22.9 | 30.8 | 8.7 | 62.3 | 40.5 | 31.0 | 21.7 | 72.6 |
| Other organ failure | | 13.1 | 11.2 | 4.8 | 9.0 | 2.3 | 61.7 | 30.5 | 42.9 | 31.4 | 63.5 |
| COPD | | 11.4 | 9.8 | 4.2 | 8.0 | 3.9 | 65.6 | 43.0 | 35.5 | 38.8 | 66.7 |
| Neurodegenerative  disease | | 17.7 | 13.1 | 7.0 | 11.9 | 5.2 | 59.8 | 30.9 | 46.3 | 29.7 | 62.6 |
| HIV/aids | | 5.9 | 5.9 | 5.9 | 2.9 | 0.0 | 32.4 | 29.4 | 8.8 | 0.0 | 32.4 |
| Nationality |  | |  |  |  |  |  |  |  |  |  |
| Belgian | 33.3 | | 30.5 | 17.1 | 23.8 | 7.0 | 62.3 | 38.4 | 34.7 | 25.2 | 69.8 |
| Non-Belgian | 30.1 | | 28.0 | 15.7 | 21.8 | 8.8 | 60.9 | 38.1 | 32.3 | 29.1 | 67.6 |
| Household type |  | |  |  |  |  |  |  |  |  |  |
| Single person household | 24.1 | | 20.9 | 12.1 | 16.4 | 4.8 | 56.0 | 31.0 | 30.9 | 23.9 | 62.2 |
| Married | 39.5 | | 36.9 | 20.8 | 28.7 | 8.7 | 66.5 | 43.7 | 36.8 | 26.2 | 75.0 |
| Living together | 34.8 | | 32.0 | 18.0 | 23.8 | 7.0 | 61.4 | 43.7 | 29.1 | 22.1 | 69.7 |
| One-parent family | 30.6 | | 27.9 | 13.8 | 22.9 | 6.9 | 62.4 | 35.4 | 37.9 | 28.4 | 68.6 |
| Other | 28.9 | | 26.3 | 12.6 | 22.2 | 4.6 | 61.9 | 29.6 | 41.4 | 28.2 | 68.5 |
| Housing comfort |  | |  |  |  |  |  |  |  |  |  |
| High | 37.7 | | 34.7 | 20.7 | 26.9 | 7.9 | 65.4 | 43.4 | 34.2 | 25.7 | 73.7 |
| Moderate | 27.3 | | 24.4 | 13.3 | 19.3 | 5.6 | 59.2 | 32.7 | 35.6 | 26.0 | 65.8 |
| Low | 32.4 | | 29.9 | 15.3 | 23.5 | 7.0 | 61.6 | 36.8 | 35.2 | 25.0 | 69.0 |
| Below low | 26.3 | | 23.7 | 12.0 | 18.7 | 6.0 | 56.4 | 30.8 | 34.8 | 24.7 | 62.7 |
| Education level |  | |  |  |  |  |  |  |  |  |  |
| No education | 31.5 | | 28.8 | 13.8 | 23.3 | 7.4 | 61.3 | 33.2 | 37.5 | 28.2 | 68.8 |
| Primary school | 31.5 | | 28.9 | 14.2 | 23.1 | 6.6 | 61.5 | 34.4 | 37.8 | 25.9 | 68.8 |
| Secondary school | 34.8 | | 31.9 | 19.0 | 24.5 | 7.3 | 62.6 | 41.1 | 32.3 | 24.6 | 70.6 |
| Post-secondary school | 37.4 | | 34.3 | 23.7 | 26.8 | 8.6 | 66.2 | 47.6 | 30.9 | 25.0 | 73.7 |
| Income level |  | |  |  |  |  |  |  |  |  |  |
| Q1 (lowest) | 35.5 | | 32.8 | 18.5 | 26.3 | 8.0 | 63.4 | 37.1 | 38.4 | 26.3 | 71.2 |
| Q2 | 31.4 | | 28.8 | 13.9 | 22.8 | 6.7 | 60.5 | 33.6 | 36.8 | 27.1 | 68.1 |
| Q3 | 32.3 | | 29.6 | 16.1 | 23.3 | 6.5 | 62.6 | 39.8 | 34.6 | 25.4 | 70.1 |
| Q4 (highest) | 33.2 | | 30.1 | 19.1 | 22.6 | 7.0 | 63.1 | 43.3 | 29.5 | 23.5 | 70.0 |
| Region |  | |  |  |  |  |  |  |  |  |  |
| Brussels-Capital region | 20.3 | | 17.3 | 15.1 | 13.2 | 5.3 | 54.5 | 36.6 | 23.1 | 25.2 | 59.6 |
| Walloon region | 29.4 | | 26.7 | 14.4 | 21.7 | 9.9 | 63.4 | 39.0 | 33.3 | 34.0 | 68.8 |
| Flemish region | 37.1 | | 34.2 | 18.8 | 26.3 | 5.7 | 62.7 | 38.4 | 37.0 | 20.7 | 71.7 |
| Degree of urbanisation | | | | |  |  |  |  |  |  |  |
| Very high | 27.0 | | 24.7 | 15.5 | 18.7 | 5.6 | 58.1 | 37.2 | 29.4 | 23.9 | 64.8 |
| High | 33.9 | | 30.8 | 18.0 | 24.0 | 6.8 | 61.7 | 38.5 | 33.9 | 24.1 | 69.6 |
| Average | 38.4 | | 35.6 | 18.3 | 28.5 | 7.5 | 66.1 | 39.7 | 40.2 | 25.0 | 74.4 |
| Low | 34.8 | | 31.9 | 16.1 | 25.2 | 9.8 | 65.3 | 38.8 | 37.0 | 32.7 | 72.2 |
| Rural | 35.7 | | 33.6 | 16.3 | 24.0 | 9.4 | 63.8 | 38.5 | 38.5 | 29.8 | 71.1 |
