# Supplementary material for: Who finds the road to palliative home care support? A nationwide analysis on the use of supportive measures for palliative home care using linked administrative databases
Source: PLoS One. 2019 Mar 12;14(3):e0213731. doi: 10.1371/journal.pone.0213731 (PMC6414004; doi:10.1371/journal.pone.0213731)
Supplement: S1 Appendix — (DOCX) [file pone.0213731.s003.docx]

**Supplementary appendix**

**Supplement to: Maetens A, Beernaert K, Deliens L, et al. Uptake of policy measures to support end-of-life home care: a population-level study using linked administrative databases.**

The authors provide this appendix to give readers additional information about their work.

**Section 1: Supplementary information on the operationalisation of sociodemographic variables**

| **Variable name** | **Data source** | **Data administrator** | **Original values (by data administrator)** | **Recoded values (by authors)** | **Description** |
| --- | --- | --- | --- | --- | --- |
| Housing comfort | Census data 2001 | Statistics Belgium | Low comfort | Low comfort | Homes that meet or exceed the following two conditions: (1) a toilet, and (2) a bathroom with bath and / or shower |
|  |  |  | No comfort | No comfort | The conditions for low comfort are not met |
|  |  |  | Moderate comfort | Moderate comfort | Homes with low comfort plus central heating |
|  |  |  | High comfort | High comfort | Homes with moderate comfort, plus a kitchen of min. 4m^2^ or a kitchen integrated into another room, plus a fixed phone line, plus one or more cars in the household |
| Education level | Census data 2011 - following the UNESCO’s International Standard Classification of Education (ISCED) | Statistics Belgium | No diploma | No diploma |  |
|  |  |  | Primary school education | Primary school education |  |
|  |  |  | Lower secondary school education | Lower secondary school education |  |
|  |  |  | Upper secondary school education | Upper secondary school education |  |
|  |  |  | Post-secondary school education (non-higher education) | Upper secondary school education |  |
|  |  |  | Higher education | Post-secondary school education |  |
|  |  |  | PhD | Post-secondary school education |  |
|  |  |  | Unknown | Missing |  |
| Household composition | Census data 2001 | Statistics Belgium | Single person household | Single person household |  |
|  |  |  | Married couple without children in the household | Married |  |
|  |  |  | Married couple with children in the household | Married |  |
|  |  |  | Couple living together without children in the household | Living together |  |
|  |  |  | Couple living together without children in the household | Living together |  |
|  |  |  | One-parent family | One-parent family |  |
|  |  |  | Collective household | Collective household |  |
|  |  |  | Other | Other |  |
| Region | Population data | InterMutualistic Agency |  | Flemish region | In total, 38 districts were recoded into three federal regions. |
|  |  |  |  | Walloon region |  |
|  |  |  |  | Brussel-capital region |  |
| Degree of urbanisation | Population data | Statistics Belgium | A | Very high | Central municipalities of the major conurbations |
|  |  |  | B1 | High | Municipalities with strong morphological and strong functional urbanisation |
|  |  |  | B2 | High | Municipalities with strong morphological and average functional urbanisation |
|  |  |  | B3 | High | Municipalities with strong morphological and weak functional urbanisation |
|  |  |  | C1 | Average | Municipalities with average morphological and strong functional urbanisation |
|  |  |  | C2 | Average | Municipalities with average morphological and average functional urbanisation |
|  |  |  | C3 | Average | Municipalities with average morphological and weak functional urbanisation |
|  |  |  | D1 | Low | Municipalities with weak morphological and strong functional urbanisation |
|  |  |  | D2 | Low | Municipalities with weak morphological and average functional urbanisation |
|  |  |  | D3 | Low | Municipalities with weak morphological and weak functional urbanisation |
|  |  |  | E | Rural | Rural communities |
| Income level | Fiscal data | Statistics Belgium | Total net taxable income (individual level) | Quartiles (aggregated) |  |
| Underlying cause of death | Death certificate data – ICD-10 code | Statistics Belgium | C00-D49 | Neoplasms |  |
|  |  |  | I11.0; I13.0; I13.2; I50 | Heart failure |  |
|  |  |  | J40-J44; J47 | Respiratory diseases |  |
|  |  |  | G10; G12.2; G20; G30; F01; F03 | Neurodegenerative diseases |  |
|  |  |  | Other | Other |  |

**Section 2: Supplementary information on the operationalisation of statutory and non-statutory supportive policy measures for palliative home care**

| **Variable name** | **Data source** | **Data administrator** | **Nomenclature code(s)** |
| --- | --- | --- | --- |
| Allowance for palliative home patients | Healthcare data | InterMutualistic Agency | 740213 |
| Multi-disciplinary support team | Healthcare data | InterMutualistic Agency | 774056, 774071, 784092 |
| Nursing care for palliative home patients | Healthcare data | InterMutualistic Agency | 427055, 427136, 427033, 427114, 427011, 427092, 427173, 427195, 427151, 427070 |
| Physiotherapy for palliative home patients | Healthcare data | InterMutualistic Agency | 564211 |
| Nursing care for heavily dependent persons | Healthcare data | InterMutualistic Agency | 425272, 425294, 425316, 425670, 425692, 425714 |
| Physiotherapy for heavily dependent persons | Healthcare data | InterMutualistic Agency | 560313, 560394, 561013, 561094, 561116, 562391, 562472, 639391, 639553, 563312, 563393, 563916, 563990, 639715 |
| Allowance for chronically ill patients | Healthcare data | InterMutualistic Agency | 740014, 740036, 740073, 740095, 740110, 740154, 740132, 740176, 740235 |

**Section 3: Hierarchical approach to the logistic regression model:**

A hierarchical approach was followed in which a first model with age, gender and cause of death as independent variables was expanded cumulatively with educational level (Model 2), household type (Model 3), housing comfort and income level (Model 4), and region and urbanisation (Model 5). This method was followed because it allows evaluating how certain groups of variables explain part of the variation. All models were checked for multicollinearity by looking at tolerance values and variance inflation factors.
